# Association between framing of the research question using the PICOT format and reporting quality of randomized controlled trials

**DOI:** 10.1186/1471-2288-10-11

**Published:** 2010-02-05

**Authors:** Lorena P Rios, Chenglin Ye, Lehana Thabane

**Affiliations:** 1 Biostatistics Unit, Father Sean O'Sullivan Research Centre, St Joseph's Healthcare, 50 Charlton Avenue East, Hamilton ON L8N 4A6, Canada; 2 Department of Clinical Epidemiology and Biostatistics, McMaster University, Hamilton ON, Canada; 3 Department of Mathematics and Statistics, McMaster University, Hamilton ON, Canada

## Abstract

**Background:**

Experts recommend formulating a structured research question to guide the research design. However, the basis for this recommendation has not been formally evaluated. The aim of this study was to examine if a structured research question using the PICOT (**P**opulation, **I**ntervention, **C**omparator, **O**utcome, **T**ime-frame) format is associated with a better reporting quality of randomized controlled trials (RCTs).

**Methods:**

We evaluated 89 RCTs reports published in three endocrinology journals in 2005 and 2006, the quality of reporting of which was assessed in a previous study. We examined whether the reports stated each of the five elements of a structured research question: population, intervention, comparator, outcome and time-frame. A PICOT score was created with a possible score between 0 and 5. Outcomes were: 1) a 14-point overall reporting quality score (OQS) based on the Consolidated Standards for Reporting Trials; and 2) a 3-point key score (KS), based on allocation concealment, blinding and use of intention-to-treat analysis. We conducted multivariable regression analyses using generalized estimating equations to determine if a higher PICOT score or the use of a structured research question were independently associated with a better reporting quality. Journal of publication, funding source and sample size were identified as factors associated with OQS in our previous report on this dataset, and therefore included in the model.

**Results:**

A higher PICOT score was independently associated with OQS (incidence rate ratio (IRR) = 1.021, 95% CI: 1.012 to 1.029) and KS (IRR = 1.142, 95% CI: 1.079 to 1.210). A structured research question was present in 33.7% of the reports and it was associated with a better OQS (IRR = 1.095, 95% CI 1.059-1.132) and KS (IRR = 1.530, 95% CI 1.311-1.786).

**Conclusions:**

Better framing of the research question using the PICOT format is independently associated with better overall reporting quality - although the effect is small - and better reporting of key methodologies.

## Background

We recently found suboptimal quality of reporting of RCTs in three general endocrinology journals and identified important deficiencies in the reporting of key methodological items [1]. Poor quality of reporting of RCTs is not limited to the endocrine literature. Similar findings have been reported for RCTs published in leading general medical journals and subspecialty journals [2-6].

Reporting deficiencies can reduce the confidence in RCT results and hinder their applications in developing clinical practice guidelines and conducting unbiased meta-analyses. The Consolidated Standards of Reporting Trials (CONSORT) group has been making efforts to improve the transparency and quality of RCT reports since 1996. They have published reporting guidelines and updates, which are available at http://www.consort-statement.org[7-9]. Journals which have adopted the CONSORT statement have shown some improvement in the quality of reporting of their manuscripts, although the magnitude of this improvement has been variable [10].

Finding predictors or determinants of quality of reporting of RCTs could guide the triage of articles that are worthwhile for busy clinicians who act as peer-reviewers. Most importantly, it could also help to improve the quality of reporting of RCTs. In our previous study, we found that journal of publication, type of funding and sample size were significantly associated with overall quality of reporting, where the assessment of quality of reporting was based on 15 out of the 22 CONSORT items [1]. We could not find any variable significantly associated with quality of reporting of key methodological items - namely, allocation concealment, blinding, and analysis by intention to treat principle [1].

A clear research question (RQ) is the starting point for any research project because it guides the development of the research design and protocol. Expert methodologists have proposed the use of a structured research question to guide this process [11]. A structured RQ about therapy should contain the following five elements: population, intervention, comparator, outcome, and time-frame. These elements are commonly referred to by the acronym PICOT [11]. There are many benefits of having a well-structured research question which include increasing the likelihood of finding a solution to the problem [12], enhancing the clarity of the thought process in developing the protocol, informing the design, guiding analysis decisions, and ensuring publication [13-15]. Whether the use of a structured RQ is associated with better quality of reporting is unknown. The aims of this study were to assess how the PICOT format is used to frame research questions, objectives or hypotheses based on reports of RCTs published in general endocrinology journals and evaluate the association between the presence of a structured RQ using the PICOT format and the quality of reporting of RCT reports.

## Methods

### Study design and setting

This is an analysis based on a systematic review of 89 RCTs published between January 2005 and December 2006 in three general endocrinology journals. We selected the three general endocrinology journals with the highest impact factor (IF) in 2006 as our source of RCT reports. These are the Journal of Clinical Endocrinology and Metabolism (IF = 5.8), Clinical Endocrinology (IF = 3.4) and the European Journal of Endocrinology (IF = 3.1). Details on how we selected the studies are described elsewhere [1]. Briefly, we included all parallel design RCTs that addressed a question of treatment or prevention. We excluded cross-over trials and trials evaluating pathophysiological mechanisms, pharmacokinetics or drug tolerability as well as economic studies and trial reports that had published their methods in a separate publication. The selection process was carried out in duplicate by two independent investigators in two screening phases: title and abstract and full text. Investigators resolved discrepancies by consensus.

### Rating the reporting quality

Full details on how we measured the reporting quality are available in our previous publication [1]. In our previous study, we chose 15 items from the revised CONSORT statement [7] to construct a 15-point overall quality score (OQS). We chose the CONSORT criteria because they are the accepted standards for reporting RCTs and they have been endorsed by many medical journals and leading editorial organizations. We defined quality of reporting as the extent to which the rationale, method, conduct and results of the trial are reported. Therefore, we adopted 15 CONSORT items pertaining to the Introduction, Methods and Results sections for our appraisal (Table 1). We chose these 15 items because lack of their reporting has been associated with higher level of bias [7]. We excluded the CONSORT discussion section items because we considered them too subjective to evaluate. We also excluded three key methodological qualities for a separate assessment. For this study, we additionally excluded the CONSORT item on the description of the objective or research question (item 6 in Table 1) as this is represented by PICOT, our explanatory variable. We scored each item 1 if it was reported and 0 if it was not clearly stated or definitely not stated. Thus, the OQS had a possible value between 0 and 14. We note here that as a study quality score, the OQS is a measure of the completeness of reporting of 14 CONSORT items.

**Table 1:** Overall Reporting Quality items

Item	Description
1. Title or Abstract	The title or the abstract states the study is a randomized controlled trial.
2. Introduction	Appropriate description of the scientific background and explanation of the rationale.
Methods:	
3. Participants	Eligibility criteria for participants are clearly described.
4. Interventions	Precise details of the interventions intended in each group are provided.
5. Outcomes	Clear definition of primary and secondary outcome measures is provided.
6. Objectives	Specific objectives or research question or hypotheses are stated.
7. Sample size	Clear description on how the sample size was determined is given.
8. Randomization sequence generation	The method used to generate the random allocation sequence is stated.
9. Randomization implementation	The separation of generator of the allocation sequence and executor is described.
10. Statistical methods	Statistical methods used to compare groups for primary outcomes, subgroup analyses or adjusted analyses were properly described.
Results:	
11. Participants flow	Number of participants randomly assigned, receiving intended treatment, completing the study protocol, and analyzed for primary outcome are given.
12. Recruitment	Dates defining the periods of recruitment and follow-up are provided.
13. Baseline data	Baseline demographic and clinical characteristics of each group are properly described.
14. Outcomes and estimation	For each primary and secondary outcome, a summary of results for each group and the estimated effect size and its precision (e.g., 95% CI) is provided.
15. Adverse events	All important adverse events or side effects in each intervention group are described.

* Item 6 (Objectives) was excluded from OQS.

We also constructed a 3-point key score (KS) based on three items that are highly important in avoiding bias: allocation concealment, blinding and analysis according to the intention to treat (ITT) principle [16,17]. We scored each item 1 if it was present and 0 if it was absent according to the definitions below. Thus, the KS had a possible value between 0 and 3.

Allocation concealment was considered to be present if one of the following allocation methods was reported: a) centralized randomization, b) numbered coded vehicles, and c) opaque, sealed and sequentially numbered envelopes. Blinding was considered to be present if at least two groups were explicitly reported as blinded. The groups considered for blinding included patients, caregivers, data collectors, outcome assessors, data analysts and manuscript writers. For studies in which blinding of patients and caregivers was considered not feasible by the reviewer, then, blinding was considered as present if at least one specific group was explicitly reported as blinded.

As the term ITT is not used consistently by researchers [18,19], we intended to capture how investigators actually conducted the analysis instead of just checking whether they stated performing an ITT analysis. For this purpose, we examined the numbers presented in the text, tables and figures of each article. We defined ITT analysis as one where all patients were analyzed as part of the group to which they were originally assigned, regardless of whether they actually satisfied the entry criteria, the treatment actually received, and subsequent protocol deviations, participant compliance or withdrawal [18].

### Rating the framing of the research question

We chose one paragraph from the introduction or methods section that best described the primary research question, hypothesis or objective. In that paragraph, we evaluated the framing of the RQ, regardless of whether it was formulated as a research question, hypothesis or objective. We examined whether the five elements of a structured RQ were present in that paragraph. The five elements were the type of patients or population relevant to the question (P), the intervention (I), the comparative intervention (C), the outcome of interest (O), and the time horizon for measuring the outcome (T). We scored each element 1 if it was present and 0 if it was absent. Thus, we created a PICOT score with a possible score between 0 and 5. The score represents a measure of the completeness of the description of the primary research question. The concept of a structured RQ was originally described involving four elements (PICO) [13] and this was probably the concept better known by investigators at the time of publication of the RCT reports under evaluation. Therefore, we decided to qualify a report as providing a structured RQ every time it described the four elements (Complete PICO) in the context of the description of the primary research question, study objective or research hypothesis. Reports that did not describe these 4 elements (Incomplete PICO) did not qualify as providing a structured RQ.

### Data abstraction

We used a standardized data abstraction form to extract data from each article. Two reviewers (LR, CY) -blinded to each other's ratings-abstracted data independently. In rating the framing of the RQ, the reviewers were blinded to the OQS and KS for each article and they resolved any disagreement through consensus. We used kappa statistics to measure inter-rater agreement for each of the five elements of the RQ. Kappa statistics for the KS items have been reported in our previous study [1] and varied from 0.55 to 0.65.

### Statistical Analysis

We calculated the percentage of trials that clearly stated each PICOT element and associated 95% confidence interval (95% CI). We used Cohen's Kappa (*κ*) statistic to calculate chance-adjusted inter-rater agreements. We judged agreement as poor if *κ *≤ 02; fair if 0.21 ≤ *κ *≤ 0.4; moderate if 0.41 ≤ *κ *≤ 0.6; substantial if 0.61 ≤ *κ *≤ 0.8; and good if *κ *>0.8 [20].

We reported descriptive statistics on categorical data as numbers (percentages) and scores (i.e., PICOT score, OQS, and KS) as median (interquartile range [IQR]).

We evaluated whether a higher PICOT score was associated with better reporting quality by conducting univariate and multivariable regression analyses with OQS and KS as the outcome variables. In our previous study on this dataset, we found three variables, i.e., publication in the JCEM, complete industrial funding and sample size, which were significantly associated with better OQS. Therefore, we included these variables in the multivariable models for OQS and KS. We used generalized estimating equations (GEE) [21] to account for the plausible correlation in the reporting quality within the same journal. We modelled within-journal correlation using an exchangeable working correlation matrix. We assumed the Poisson distribution for outcomes in GEE, as rating scores are non-negative counts. The results were reported as exponents of the coefficient estimates of the GEE analysis, which represent the incidence rate ratios (IRR) over the period of interest - January 2005 to December 2006. Using the same statistical approach as above, we also conducted univariate and multivariable regression analyses to determine whether the use of a structured RQ (complete PICO) was associated with a better OQS and KS. Variables were considered to be statistically significant at alpha = 0.05. We conducted all analyses using SAS 9.0 (Cary, NC).

## Results

### Framing of the research question

For the rating of the individual components of the RQ, the *κ *inter-rater agreement estimate was 0.54 (95% CI: 0.32, 0.77) for patients, 0.52 (95% CI: 0.15, 0.88) for intervention, 0.87 (95% CI: 0.59, 0.87) for comparator, 0.20 (95% CI: 0.10, 0.30) for outcome, and 0.60 (95% CI: 0.41, 0.78) for time-frame. The median PICOT score was 3 (IQR = 1). The percentage of articles that reported each element of the primary RQ is provided on Table 2. Patients, intervention and outcome were often adequately described. However, half of the reports did not describe the comparison intervention and a minority described the study time frame. A complete description of an ideal PICOT RQ was present in 16 out of the 89 reports (18.0%). A structured RQ (Complete PICO) was present in 30 reports (33.71%).

**Table 2:** Frequency of description of each PICOT element

	All articles n = 89		
Element of the research question	n	%	95% CI
**P: Patients**	73	82	74 - 90
**I: Intervention**	83	93	88 - 98
**C: Comparator**	47	52	42 - 63
**O: Outcome**	65	73	63 - 82
**T: Time**	26	29	19- 38
**Complete PICOT**	16	18	10 - 27
**Complete PICO**	30	33	24 - 44

CI: Confidence interval

### Association between framing of the research question and reporting quality

Tables 3 and 4 display the results of the univariate and multivariable analyses of factors associated with OQS and KS, respectively. A higher PICOT score was significantly associated with a higher OQS (Table 3) and KS (Table 4) in both univariate and multivariable analyses. After adjusting for journal of publication, sample size and funding source, each point increase in PICOT score was significantly associated with a 2.1% increase in the OQS and a 14.2% increase in the KS.

**Table 3:** Association between PICOT score and overall reporting quality

Independent Variable	Median	Q1-Q3	Multivariable Model			Univariate Models		
			
	OQS^a^		IRR	95% CI	p value	IRR	95% CI	p value
**PICOT**	9	8 - 10	1.021^b^	1.012 - 1.029	< 0.0001	1.018	1.006 - 1.029	0.0032
								
**Funding Source**								
Others^c^	9	8 - 11	1	-	-	1	-	-
Completely funded by industry	10	9 - 10	1.022	0.999 - 1.046	0.0638	1.058	1.010 - 1.108	0.0177
								
**Journal of Publication**								
Others^c^	9	7 - 10	1	-	-	1	-	-
J Clin Endocrinol Metab	9	8 - 11	1.055	0.985 - 1.130	0.1280	1.074	0.986 - 1.171	0.1027
								
**Sample Size**	9	8 - 10	1.073^d^	1.026 - 1.123	0.0020	1.095	1.011 - 1.185	0.0250

Abbreviations: OQS, Overall quality score; Q1, quartile 1; Q3, quartile 3; IRR, incidence rate ratio; 95% CI, 95% confidence interval; GEE, generalized estimating equations; RCTs, randomized controlled trials.

^a ^Maximum possible score = 14

^b ^The value is an expression of the change in the average of the OQS due to one unit increase in PICOT score.

^c ^Reference category

^d ^The sample size variable was log(10) transformed. The value is an expression of the change in the average of the OQS due to one unit increase in sample size in the log scale.

**Table 4:** Association between PICOT score and reporting of key quality elements

Independent Variable	Median	Q1-Q3	Multivariable Model			Univariate Models		
			
	KS^a^		IRR	95% CI	p value	IRR	95% CI	p value
**PICOT**	0	0 - 1	1.142^b^	1.079 - 1.210	< 0.0001	1.142	1.058 - 1.232	0.0006
								
**Funding Source**								
Others^c^	0	0 - 1	1	-	-	1	-	-
Completely funded by industry	1	0 - 1	1.074	0.747 - 1.544	0.7013	1.112	0.761 - 1.623	0.5834
								
**Journal of Publication**								
Others^c^	0	0 - 1	1	-	-	1	-	-
J Clin Endocrinol Metab	0	0 - 1	0.881	0.533 - 1.455	0.6205	0.924	0.515 - 1.658	0.7919
								
**Sample Size**	0	0 - 1	1.255^d^	0.936 - 1.684	0.1292	1.222	0.922 - 1.619	0.1623

Abbreviations: KS, Key score; Q1, quartile 1; Q3, quartile 3; IRR, incidence rate ratio; 95% CI, 95% confidence interval; GEE, generalized estimating equations; RCTs, randomized controlled trials.

^a ^Maximum possible score = 3

^b ^The value is an expression of the change in the average of the key score due to one unit increase in PICOT score.

^c ^Reference category

^d ^The sample size variable was log(10) transformed. The value is an expression of the change in the average of the key score due to one unit increase in sample size in the log scale.

As shown on Tables 5 and 6, the use of a structured RQ (complete PICO) was also significantly associated with better overall reporting quality (Table 5) and better reporting of key quality elements (Table 6). After adjusting for confounding variables, the presence of a structured RQ was associated with a 9.5% increase in the OQS and a 53.0% increase in KS. The association between the reporting of individual PICOT elements and OQS is available as an additional file (additional file 1).

**Table 5:** Association between a structured research question and overall reporting quality

Independent Variable	Median	Q1-Q3	Multivariable Model			Univariate Models		
			
	OQS1^a^		IRR	95% CI	p value	IRR	95% CI	p value
**Structured RQ**								
Not complete PICO^c^	9	7 - 10	1	-	-	1	-	-
Complete PICO	10	9 - 11	1.095	1.059 - 1.132	< 0.0001	1.104	1.084 - 1.125	< 0.0001
								
**Funding Source**								
Others^c^	9	8 - 11	1	-	-	1	-	-
Completely funded by industry	10	9 - 10	1.256	1.006 - 1.046	0.0113	1.074	0.986 - 1.171	0.1027
								
**Journal of Publication**								
Others^c^	9	7 - 10	1	-	-	1	-	-
J Clin Endocrinol Metab	9	8 - 11	1.039	0.962 - 1.121	0.3325	1.074	0.986 - 1.171	0.1027
								
**Sample Size**	9	8 - 10	1.066^d^	1.012 - 1.123	0.0169	1.095	1.011 - 1.185	0.0250

Use of a structured RQ is defined as the reporting of complete PICO

Abbreviations: OQS, Overall quality score; Q1, quartile 1; Q3, quartile 3; IRR, incidence rate ratio; 95% CI, 95% confidence interval; GEE, generalized estimating equations; RCTs, randomized controlled trials.

^a ^Maximum possible score = 14

^b ^The value is an expression of the change in the average of the OQS due to one unit increase in PICOT score.

^c ^Reference category

^d ^The sample size variable was log(10) transformed. The value is an expression of the change in the average of the OQS due to one unit increase in sample size in the log scale.

**Table 6:** Association between a structured research question and reporting of key quality elements

Independent Variable	Median	Q1-Q3	Multivariable Model			Univariate Models		
			
	KS^a^		IRR	95% CI	p value	IRR	95% CI	p value
**Structured RQ**								
Not complete PICO^c^	0	0 - 1	1	-	-	1	-	-
Complete PICO	1	0 - 1	1.530	1.311 - 1.786	< 0.0001	1.510	1.398 - 1.632	< 0.0001
								
**Funding Source**								
Others^c^	0	0 - 1	1	-	-	1	-	-
Completely funded by industry	1	0 - 1	1.088	0.775 - 1.528	0.6249	1.112	0.761 - 1.623	0.5834
								
**Journal of Publication**								
Others^c^	0	0 - 1	1	-	-	1	-	-
J Clin Endocrinol Metab	0	0 - 1	0.808	0.466 - 1.400	0.4473	0.924	0.515 - 1.658	0.7919
								
**Sample Size**	0	0 - 1	1.216^d^	0.876 - 1.689	0.2421	1.222	0.922 - 1.619	0.1623

Use of a structured RQ is defined as the reporting of complete PICO

Abbreviations: KS, Key score; Q1, quartile 1; Q3, quartile 3; IRR, incidence rate ratio; 95% CI, 95% confidence interval; GEE, generalized estimating equations; RCTs, randomized controlled trials.

^a ^Maximum possible score = 3

^b ^The value is an expression of the change in the average of the key score due to one unit increase in PICOT score.

^c ^Reference category

^d ^The sample size variable was log(10) transformed. The value is an expression of the change in the average of the key score due to one unit increase in sample size in the log scale.

## Discussion

We evaluated the prevalence of the use of the PICOT format in framing the RQ in a sample of articles published in three general endocrinology journals in 2005 and 2006. The framing of the RQ was usually incomplete and unclear, with only one-third of the reports using a structured approach based on the PICOT format. These observations are consistent with a recent survey of four anesthesia journals, which found that 96% of the studies did not fully apply the PICOT approach in reporting the research question [13].

To the best of our knowledge, this is the first study assessing the association between the framing of the RQ and RCT reporting quality. Our results consistently indicate a significant association between the completeness of the RQ description and quality of reporting. We found that the presence of a structured RQ is significantly associated with a 9.5% increase in the OQS and a 53.0% increase in KS.

A bigger sample size, complete industry funding and publication in the Journal of Clinical Endocrinology and Metabolism were also significantly associated with overall reporting quality but not with the report of key methodological items. It is plausible that part of the variation of the quality of reporting between RCT reports can be explained by other variables such as awareness of the CONSORT statement by authors, adoption of CONSORT by journals and availability of advice from a methodological expert when planning an RCT. However, testing these hypotheses was out of the scope of our study.

The use of a structured RQ has been proposed as a systematic way to construct the study question to aid the search for valid answers [22]. In general, a structured RQ can guide the literature search, protocol development and the conduct of a study. The explicit statement of the five PICOT elements prompts the investigator to think about the design to use and to consider the balance between the research question and the feasibility to answer it [22]. This also forms the basis for the recommendation by experts in clinical epidemiology to use a structured approach when formulating research questions [11]. The Cochrane collaboration also advocates the same approach in formulating research questions for their systematic reviews [23].

There are several limitations to our study. First, we did not measure RCT methodological quality directly, as we did not verify the information from the authors or their protocols. Therefore, the quality of reporting should be taken only as a surrogate of true methodological quality since important methodological detail may be omitted from published reports[24,25]. In addition, some of the items of the OQS explicitly assessed the completeness of reporting - as a measure of reporting quality-rather than the adequacy of the methods. On the other hand, the KS can be considered a better indicator of methodological quality since the three elements directly relate to the adequacy of the methods used in each trial. The presence of a stronger association with KS as compared to OQS suggests that the use of a structured RQ could be associated with better methodological quality. Second, our reporting quality scores are not validated. There are more than 25 quality assessment scales, but most of them have not been rigorously tested for validity and reliability [26]. Our OQS score is mainly a measure of the completeness of reporting. We based our score on the CONSORT criteria because they are the most accepted standards for reporting RCTs and have been widely endorsed by many clinical journals and editorial organizations. Third, our analyses rely on quality scores, which can be problematic [27-31]. Problems with scales relate to both the choice of elements to include in a score and how these elements should be weighted [28,29]. Different methods to create the scores may lead to different results when the scores are used in a particular analysis. Several studies have shown a lack of agreement between scores or scales in separating studies into low and high quality and no scale has been found to be the best at validly measuring quality [27,29,31]. This suggests that different scales are probably measuring different constructs and it can be difficult to assign a meaning to a particular quality score. Therefore, evaluating the quality of RCTs for systematic reviews by analyzing quality items individually is often considered a more preferred approach than relying on combining the information in a single numerical value. This approach may allow assigning different levels of importance to individual quality items depending on the context of the particular trial [28]. To avoid the limitations of using quality scores, we could have conducted an analysis of the association between the use of a structured RQ and the reporting of each individual key element. However, our study lacked of statistical power for such analysis as key methodologies were infrequently reported. Therefore, we opted for using the OQS and the KS. Caution should therefore be taken in assigning a specific meaning to each of our scores. The OQS is merely a measure of completeness of reporting. The KS combines the information on the use of allocation concealment, blinding and intention to treat analysis. These items have been shown to be associated with bias [16,17]. Fourth, the framing of PICOT is itself an aspect of reporting quality. To avoid this problem, at least in part, we excluded the item related to description of the objectives or RQ from our OQS. Finally, the inclusion of only general endocrinology journals may affect the generalizability of our results. Our findings would need to be confirmed by a similar and larger study applied to a broader sample of RCT reports in other specialties and also in leading general medical journals. In spite of these limitations, we think our results have good internal validity. We used a standardized evaluation instrument, two reviewers independently performed the selection and abstraction processes, and disagreements were always resolved by consensus.

## Conclusions

Our study findings show that the use of the PICOT format to structure the RQ in RCT reports published in general endocrinology journals over 2005-2006 was low. We also found a small association between the use of a structured RQ based on the PICOT format and a better overall reporting quality of RCTs. The effect on key methodologies was more pronounced. It is important to recognize that while poor reporting does not necessarily mean poor design or conduct of a study, the quality of reporting is routinely used by researchers as a proxy for study quality in systematic reviews. An examination of a broader sample of studies, including other areas of medicine, would be necessary to confirm our results. The main implication of this study is that the researchers should pay attention to proper framing of the research question - they should consider using a structured approach such as the PICOT format to frame it as this is likely to determine how the study is designed, conducted and ultimately reported.

## Competing interests

The authors declare that they have no competing interests.

## Authors' contributions

LR designed the study, carried out data abstraction, participated in the interpretation of data and drafted the manuscript. CY carried out data abstraction, performed the statistical analysis and revised the manuscript. LT conceived the study, participated in the interpretation of data and critical revision of the manuscript. All authors read and approved the final manuscript.

## Authors Information

LT is a clinical trials mentor for the Canadian Institutes of Health Research.

## Pre-publication history

The pre-publication history for this paper can be accessed here:

http://www.biomedcentral.com/1471-2288/10/11/prepub

## Supplementary Material

Additional file 1 **Association between the reporting of individual PICOT elements and overall reporting quality (OQS)**. The table shows the association between the reporting of each individual PICOT element and OQS expressed as incidence rate ratio (IRR). In the multivariable analysis, there was a statistically significant negative association between the reporting of the intervention and the comparator in the research question and OQS. Conversely, there was a statistically significant positive association between the reporting of the time frame in the research question and OQS. The magnitude of all these associations was small. Click here for file

## References

[B1] Rios LP Odueyungbo A Moitri MO Rahman MO Thabane L Quality of reporting of randomized controlled trials in general endocrinology literature J Clin Endocrinol Metab 2008 93 3810 3816 10.1210/jc.2008-0817 18583463

[B2] Altman DG The scandal of poor medical research BMJ 1994 308 283 284 8124111 10.1136/bmj.308.6924.283 PMC2539276

[B3] Balasubramanian SP Wiener M Alshameeri Z Tiruvoipati R Elbourne D Reed MW Standards of reporting of randomized controlled trials in general surgery: can we do better? Ann Surg 2006 244 663 667 10.1097/01.sla.0000217640.11224.05 17060756 PMC1856614

[B4] Dias S McNamee R Vail A Evidence of improving quality of reporting of randomized controlled trials in subfertility Hum Reprod 2006 21 2617 2627 10.1093/humrep/del236 16793995

[B5] Mills E Loke YK Wu P Montori VM Perri D Moher D Guyatt G Determining the reporting quality of RCTs in clinical pharmacology Br J Clin Pharmacol 2004 58 61 65 10.1111/j.1365-2125.2004.2092.x 15206994 PMC1884547

[B6] Scales CD Jr Norris RD Keitz SA Peterson BL Preminger GM Vieweg J Dahm P A critical assessment of the quality of reporting of randomized, controlled trials in the urology literature J Urol 2007 177 1090 1094 10.1016/j.juro.2006.10.027 17296417

[B7] Altman DG Schulz KF Moher D Egger M Davidoff F Elbourne D Gotzsche PC Lang T Consort G The revised CONSORT statement for reporting randomized trials: explanation and elaboration Ann Intern Med 2001 134 663 694 11304107 10.7326/0003-4819-134-8-200104170-00012

[B8] Begg C Cho M Eastwood S Horton R Moher D Olkin I Pitkin R Rennie D Schulz KF Simel D Stroup DF Improving the quality of reporting of randomized controlled trials. The CONSORT statement JAMA 1996 276 637 639 10.1001/jama.276.8.637 8773637

[B9] Moher D Schulz KF Altman DG The CONSORT statement: revised recommendations for improving the quality of reports of parallel-group randomized trials Ann Intern Med 2001 134 657 662 11304106 10.7326/0003-4819-134-8-200104170-00011

[B10] Plint AC Moher D Morrison A Schulz K Altman DG Hill C Gaboury I Does the CONSORT checklist improve the quality of reports of randomised controlled trials? A systematic review Med J Aust 2006 185 263 267 16948622 10.5694/j.1326-5377.2006.tb00557.x

[B11] Haynes R Haynes R, Sacket D, Guyatt G, Tugwell P Forming research questions Clinical Epidemiology: How to do Clinical Practice Research 2006 3 Philadelphia, PA: Lippincott Williams & Wilkins 3 14

[B12] Clouse RE Proposing a good research question: a simple formula for success Gastrointest Endosc 2005 61 279 280 10.1016/S0016-5107(04)02579-9 15729240

[B13] Thabane L Thomas T Ye C Paul J Posing the research question: not so simple Can J Anesth 2009 56 71 79 10.1007/s12630-008-9007-4 19247780

[B14] Sackett DL Wennberg JE Choosing the best research design for each question BMJ 1997 315 1636 9448521 10.1136/bmj.315.7123.1636 PMC2128012

[B15] Stone P Deciding upon and refining a research question Palliat Med 2002 16 265 267 10.1191/0269216302pm562xx 12047007

[B16] Moher D Pham B Jones A Cook DJ Jadad AR Moher M Tugwell P Klassen TP Does quality of reports of randomised trials affect estimates of intervention efficacy reported in meta-analyses? Lancet 1998 352 609 613 10.1016/S0140-6736(98)01085-X 9746022

[B17] Schulz KF Chalmers I Hayes RJ Altman DG Empirical evidence of bias. Dimensions of methodological quality associated with estimates of treatment effects in controlled trials JAMA 1995 273 408 412 10.1001/jama.273.5.408 7823387

[B18] Hollis S Campbell F What is meant by intention to treat analysis? Survey of published randomised controlled trials BMJ 1999 319 670 674 10480822 10.1136/bmj.319.7211.670 PMC28218

[B19] Montori VM Guyatt GH Intention-to-treat principle CMAJ 2001 165 1339 1341 11760981 PMC81628

[B20] Landis JR Koch GG The measurement of observer agreement for categorical data Biometrics 1977 33 159 174 10.2307/2529310 843571

[B21] Zeger SL Liang KY Albert PS Models for longitudinal data: A generalized estimating equation approach Biometrics 1988 44 1049 1060 10.2307/2531734 3233245

[B22] Haynes B Forming research questions J Clin Epidemiol 2006 59 881 886 10.1016/j.jclinepi.2006.06.006 16895808 PMC7125967

[B23] O'Connor D Green S Higgins JPT Higgins JPT, Green S Defining the review question and developing criteria for including studies Cochrane handbook for systematic reviews of interventions 2008 Chichester: John Wiley & Sons Ltd 83 94

[B24] Devereaux PJ Choi PT El-Dika S Bhandari M Montori VM Schunemann HJ Garg AX Busse JW Heels-Ansdell D Ghali WA Manns BJ Guyatt G An observational study found that authors of randomized controlled trials frequently use concealment of randomization and blinding, despite the failure to report these methods J Clin Epidemiol 2004 57 1232 1236 10.1016/j.jclinepi.2004.03.017 15617948

[B25] Pildal J Chan AW Hrobjartsson A Forfang E Altman DG Gotzsche PC Comparison of descriptions of allocation concealment in trial protocols and the published reports: cohort study BMJ 2005 330 1049 10.1136/bmj.38414.422650.8F 15817527 PMC557221

[B26] Olivo SA Macedo LG Gadotti IC Fuentes J Stanton T Magee DJ Scales to Assess the Quality of Randomized Controlled Trials: A Systematic Review Phys Ther 2008 88 156 175 18073267 10.2522/ptj.20070147

[B27] Juni P Witschi A Bloch R Egger M The hazards of scoring the quality of clinical trials for meta-analysis JAMA 1999 282 1054 1060 10.1001/jama.282.11.1054 10493204

[B28] Juni P Altman DG Egger M Systematic reviews in health care: Assessing the quality of controlled clinical trials BMJ 2001 323 42 46 10.1136/bmj.323.7303.42 11440947 PMC1120670

[B29] Whiting P Harbord R Kleijnen J No role for quality scores in systematic reviews of diagnostic accuracy studies BMC Med Res Methodol 2005 5 19 10.1186/1471-2288-5-19 15918898 PMC1184082

[B30] Juni P Egger M Scoring the quality of clinical trials JAMA 2000 283 1422 1423 10732924

[B31] Herbison P Hay-Smith J Gillespie WJ Adjustment of meta-analyses on the basis of quality scores should be abandoned J Clin Epidemiol 2006 59 1249 1256 10.1016/j.jclinepi.2006.03.008 17098567

