# Supplementary material for: Association between framing of the research question using the PICOT format and reporting quality of randomized controlled trials
Source: BMC Med Res Methodol. 2010 Feb 5;10:11. doi: 10.1186/1471-2288-10-11 (PMC2834676; doi:10.1186/1471-2288-10-11)
Supplement: Additional file 1 — Association between the reporting of individual PICOT elements and overall reporting quality (OQS). The table shows the association between the reporting of each individual PICOT element and OQS expressed as incidence rate ratio (IRR). In the multivariable analysis, there was a statistically significant negative association between the reporting of the intervention and the comparator in the research question and OQS. Conversely, there was a statistically significant positive association between the reporting of the time frame in the research question and OQS. The magnitude of all these associations was small. [file 1471-2288-10-11-S1.DOC]

**Association between individual PICOT elements and OQS**

| **PICOT Element** ^a^ | **Univariate Models** | | | **Multivariable Model** ^b^ | | |
| --- | --- | --- | --- | --- | --- | --- |
|  | **IRR** | **95% CI** | **p value** | **IRR** | **95% CI** | **p value** |
| **P – Population** |  |  |  |  |  |  |
| Clearly addressed ^c^ | 1 | - | - | 1 | - | - |
| Not clearly addressed | 0.989 | 0.903 – 1.084 | 0.8158 | 0.986 | 0.934 – 1.039 | 0.5922 |
|  |  |  |  |  |  |  |
| **I – Intervention** |  |  |  |  |  |  |
| Clearly addressed ^c^ | 1 | - | - | 1 | - | - |
| Not clearly addressed | 0.941 | 0.940 – 0.942 | < 0.0001 | 0.990 | 0.987 – 0.994 | < 0.0001 |
|  |  |  |  |  |  |  |
| **C - Comparator** |  |  |  |  |  |  |
| Clearly addressed ^c^ | 1 | - | - | 1 | - | - |
| Not clearly addressed | 0.879 | 0.834 – 0.930 | < 0.0001 | 0.873 | 0.818 – 0.931 | < 0.0001 |
|  |  |  |  |  |  |  |
| **O - Outcome** |  |  |  |  |  |  |
| Clearly addressed ^c^ | 1 | - | - | 1 | - | - |
| Not clearly addressed | 0.980 | 0.905 – 1.061 | 0.6154 | 0.995 | 0.923 – 1.073 | 0.9030 |
|  |  |  |  |  |  |  |
| **T – Time Frame** |  |  |  |  |  |  |
| Clearly addressed ^c^ | 1 |  |  | 1 | - | - |
| Not clearly addressed | 0.991 | 0.977 – 1.006 | 0.2342 | 1.035 | 1.020 – 1.050 | < 0.0001 |

^a^ Adjusted for confounders: Funding source, Journal of publication and Sample size.

^b^ Multi-collinearity was not detected in any PICOT element; the variance inflation factor (VIF) was less than 1.2 for every element.

^c^ Reference category
